# Isoquercitrin Alleviates 5‐Fluorouracil‐Induced Intestinal Mucositis in Mice by Modulating Inflammation and Oxidative Stress

**DOI:** 10.1002/cbdv.202503827

**Published:** 2026-04-23

**Authors:** Lázaro de Sousa Fideles, Matheus da Silva Campelo, João Francisco Câmara Neto, Conceição da Silva Martins, João Erivan Façanha Barreto, Ícaro Gusmão Pinto Vieira, Nágila Maria Pontes Silva Ricardo, Gilberto Santos Cerqueira, Maria Elenir Nobre Pinho Ribeiro

**Affiliations:** ^1^ Center For Studies in Microscopy and Image Processing, Faculty of Medicine, Department of Morphology Federal University of Ceará Porangabuçu Campus Fortaleza Ceará Brazil; ^2^ Laboratory of Polymers and Materials Innovation, Center of Sciences, Department of Organic and Inorganic Chemistry Federal University of Ceará Pici Campus Fortaleza Ceará Brazil; ^3^ Technological Development Park Federal University of Ceará Pici Campus Fortaleza Ceará Brazil

**Keywords:** 5‐fluorouracil, intestinal mucositis, isoquercitrin, natural products

## Abstract

This study aimed to evaluate the role of isoquercitrin (ISO) in 5‐fluorouracil‐induced intestinal mucositis. The animals (6 animals/group) were divided into: vehicle (2% DMSO), 5‐fluorouracil (5‐FU) and groups receiving ISO (10, 50, or 100 mg/kg). ISO at 100 mg/kg (ISO‐100) (232.5 ± 3.8 µm) attenuated the reduction in villus height induced by 5‐FU (125.3 ± 1.5 µm) and the reduction in crypt depth in the intestine (ISO‐100: 101.7 ± 3.5 µm vs 5‐FU: 70.8 ± 1.6 µm; p < 0.05). The jejunum was chosen for assays involving oxidative stress and inflammation. ISO‐100 attenuated the mastocytosis (ISO‐100: 7.6 ± 0.1 mast cells/field vs 5‐FU: 12.5 ± 0.2 mast cells/field; *p* < 0.05) and goblet cell depletion (ISO‐100: 11.0 ± 0.5 cells/field vs 5‐FU: 8.0 ± 0.6 cells/field; *p* < 0.05). The treatment with ISO‐100 showed lower levels of MDA and MPO, and increased GSH levels. Administration of celecoxib (selective COX‐2 inhibitor) + ISO‐100 increased the villus height and the immunohistochemistry showed that ISO‐100 + CLX (7.4% ± 0.1%) attenuates the expression of COX‐2 in jejunum induced by 5‐FU (22.5% ± 0.1%). These results show that ISO‐100 attenuates 5‐FU‐induced intestinal mucositis by modulating the inflammatory process and reducing oxidative stress.

## Graphical abstract

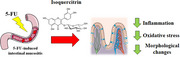

## 1 Introduction

Chemotherapy remains a central pillar in cancer therapy, administered either alone or in combination with complementary approaches to optimize therapeutic outcomes. In addition to classic cytotoxic agents, targeted therapy has introduced agents that target specific molecular alterations, selected through biomarker profiling [1]. The available drugs encompass mechanistically distinct classes: alkylating agents, antimetabolites, topoisomerase inhibitors, microtubule poisons, and platinum compounds, but they largely converge on nucleic acid damage and apoptotic cell death [2].

Although designed to act preferentially on neoplastic cells, these agents also affect rapidly renewing tissues, such as gastrointestinal mucosa, hair follicles, and bone marrow, leading to adverse events that can interrupt treatment and compromise outcomes [3]. Toxicities may arise early, at intermediate times, or late, and include nausea, mucositis, myelosuppression, alopecia, and neurotoxicity, among others [4].

5‐Fluorouracil (5‐FU) is a uracil‐derived antimetabolite that remains widely used in cancer chemotherapy. Its activity depends on conversion into active metabolites (FdUMP, FdUTP, FUTP) that interfere with thymidylate synthesis, incorporate into DNA/RNA, and trigger cytotoxicity; additional effects include disruption of essential tRNA/rRNA modifications and ribosomal collisions during translation, culminating in cellular stress and apoptosis [5].

Clinically, 5‐FU is employed in the treatment of breast, colon, and lung cancers, but it can cause neurotoxicity, cardiotoxicity, hepatotoxicity, and myelotoxicity, as well as skin reactions and gastrointestinal mucosal lesions [6]. In this context, intestinal mucositis is a frequent complication characterized by inflammation, erosions, and ulcerations throughout the digestive tract, leading to pain, feeding dysfunction, diarrhea, and increased risk of secondary infections [7].

The canonical pathophysiology of mucositis unfolds through five interconnected stages. It begins with the initiation phase, characterized by direct DNA damage and the generation of reactive oxygen species. Subsequently, signaling pathways such as p53 and NF‐κB are activated, triggering the early cellular response. The process is then amplified by the release of inflammatory mediators, which perpetuate tissue injury and ultimately culminate in mucosal ulceration [8]. Several mediators drive this inflammatory cascade, since injured mucosal tissue shows increased expression of pro‐inflammatory cytokines, including TNF‐α, IL‐1β, and IL‐6, as well as activate NF‐κB, induce endothelial adhesion molecules and amplify leukocyte recruitment [9, 10].

Inducible COX‐2, positively regulated by cytokines, contributes to the persistence of inflammation and correlates with the severity of mucositis [11]. Furthermore, oxidative stress and inflammatory biomarkers are widely used to monitor damage and test interventions, such as reduced glutathione (GSH), malondialdehyde (MDA), and myeloperoxidase (MPO) [12]. The increase in levels of pro‐inflammatory markers associated with depletion of endogenous anti‐inflammatory agents is linked to a worse prognosis for mucositis, as it leads to increased loss of tissue microstructure. In addition, maintaining this “inflammatory status” and increasing oxidative stress amplifies the severity of the lesions, which delays the tissue repair [13].

Based on this, natural products, particularly flavonoids have emerged as promising adjuvants for intestinal mucositis treatment due to their antioxidant, anti‐inflammatory, and cytoprotective properties [14]. Isoquercitrin (quercetin‐3‐O‐glycoside) (ISO), abundant in species such as *Dimorphandra gardneriana* has a broad pharmacological profile: it neutralizes ROS/RNS, increases SOD, CAT, GSH, and reduces COX‐2, iNOS, and pro‐inflammatory cytokines through modulation of TLR4/NF‐κB and MAPK pathways [15]. In the intestine, flavonoids help maintain epithelial barrier integrity and demonstrates therapeutic potential through its antioxidant, anti‐inflammatory, and cytoprotective effects [16]. Despite these effects, there are no reported data in the literature regarding the protective effect of ISO against preclinical models of mucositis, whether intestinal or oral.

Based on this, the present study aimed to evaluate the protective effect of ISO against the 5‐fluorouracil‐induced intestinal mucositis in mice, by assessing its effects on the levels of mediators linked to oxidative stress (reduced glutathione and malondialdehyde) and inflammation (myeloperoxidase and cyclooxygenase‐2), as well as intestinal microarchitecture (number of mast cells, goblet cells, villi and crypts).

## 2 Materials and Methods

### 2.1 Materials

For the experiments, 5‐fluorouracil (5‐FU—FauldFluor, Libbs, São Paulo, Brazil) and celecoxib (CLX—Celebra, Pfizer, São Paulo, Brazil) was used. Isoquercitrin (ISO) was isolated from *Dimorphandra gardneriana*. The plant material was collected in Crato (Ceará—Brazil), northeastern Brazil (semi‐arid climate), between July and August. The aerial parts of the plant were used to confirm its authenticity, and the exsiccata was deposited in the Prisco Bezerra Herbarium of the Federal University of Ceará under protocol number 32339. The collected specimen has been duly catalogued in the National System for the Management of Genetic Heritage and Associated Traditional Knowledge, ensuring compliance with current Brazilian biodiversity regulations (SisGen) under the code AC29F45. The isolation method and structural characterization data for ISO are reported in the Supporting Information. The other reagents are of analytical grade.

### 2.2 Methods

#### 2.2.1 Animals

For this study, male Swiss mice weighing between 25‐30 g were used (6 animals/group). The mice were kept in cages containing a thin layer of wood shavings, as well as food and water *ad libitum*. The environment was maintained under controlled temperature (23% ± 2°C) and light (12 h light/12 h dark cycle) conditions. All procedures described were performed after approval of the protocols by the Animal Use Ethics Committee of the Federal University of Ceará, under protocol number 1010230921.

#### 2.2.2 Experimental Protocol

As displayed in Table 1, a description of the different experimental groups used in this study. For induction of intestinal mucositis, on the first day a single intraperitoneal (i.p.) dose of 5‐fluorouracil (5‐FU; 450 mg/kg) was administered to initiate the injury process [17]. A 2% DMSO (v/v) solution was used as a negative control (vehicle) due to the low solubility of isoquercitrin (ISO) in aqueous medium. It is important to mention that the use of DMSO at this concentration presents low acute toxicity via the oral route and is commonly used in intestinal mucositis models to solubilize hydrophobic drugs. These statements can be verified by analyzing the histopathological micrographs obtained for the vehicle group, in which tissue integrity is observed [15].

**TABLE 1:** Experimental groups used in evaluating the protective potential of isoquercitrin against the 5‐FU‐induced intestinal mucositis model in mice.

Groups (*n* = 6)	Description
Vehicle	Gavage with 2% DMSO (v/v)
5‐FU	Gavage with 2% DMSO (v/v) + 5‐FU at 450 mg/kg (i.p.)
ISO‐10	Gavage with ISO at 10 mg/kg + 5‐FU at 450 mg/kg (i.p.)
ISO‐50	Gavage with ISO at 50 mg/kg + 5‐FU at 450 mg/kg (i.p.)
ISO‐100	Gavage with ISO at 100 mg/kg + 5‐FU at 450 mg/kg (i.p.)
CLX	CLX at 7.5 mg/kg (i.p.) + 5‐FU at 450 mg/kg (i.p.)
ISO + CLX	CLX at 7.5 mg/kg (i.p.) + Gavage with ISO at 100 mg/kg + 5‐FU at 450 mg/kg (i.p.)

Where DMSO: dimethyl sulfoxide; 5‐FU: 5‐fluorouracil; ISO: isoquercitrin and CLX: celecoxib.

The administration of ISO began with an initial dose given one hour before the 5‐FU injection. The subsequent doses were administrated 24 and 48 h after 5‐FU exposure. The experimental protocol therefore considered both a pre‐treatment and post‐treatment regimen in order to evaluate the potential of ISO in preventing, as well as mitigating, the harmful effects induced by 5‐FU. The animals received ISO at 10, 50 or 100 mg/kg (p.o.), according data reported in previous investigations [18, 19]. On the fourth experimental day, the euthanasia was performed using an overdose of xylazine (30 mg/kg, i.p.) and ketamine (300 mg/kg, i.p.) and intestinal tissues were promptly excised for further analysis.

To clarify the role of cyclooxygenase‐2 (COX‐2) in the protective effect of ISO during 5‐FU‐induced intestinal mucositis, COX‐2 activity was selectively blocked with celecoxib administration (CLX, 7.5 mg/kg, i. p.). Using the optimal ISO dose previously established (100 mg/kg), the 5‐FU‐induced mucositis protocol was replicated, maintaining the same experimental design as in the initial phase. The animals were randomly assigned to three experimental groups: one administered isoquercitrin (ISO 100) orally at 100 mg/kg; another receiving intraperitoneal celecoxib (CLX, 7.5 mg/kg); and a third group treated with the combined regimen of both agents (ISO 100 mg/kg, p.o. + CLX 7.5 mg/kg, i.p.) [20].

#### 2.2.3 Parameters Evaluated

##### 2.2.3.1 Histopathology and Morphometry

Following euthanasia, intestinal samples were carefully excised from specific anatomical regions: the duodenum located approximately 3 cm upstream of the ligament of Treitz; the jejunum, positioned about 10 cm above the same reference point; and the ileum, situated roughly 6 cm from the ileocecal valve. Intestinal samples were immersed in 10% formalin for 24 h to ensure proper tissue fixation, subsequently processed by standard histological techniques, and stained with hematoxylin‐eosin (H&E) for microscopic evaluation.

The severity of intestinal mucositis was studied by a pathologist, in a blinded manner, who analyzed all prepared slides and assigned histopathological scores to the degree of damage in the intestinal microenvironment, where: 0 (absence), 1 (low), 2 (moderate) and 3 (severe) [21]. The assignment of histopathological scores took into account the following aspects: villus shortening, epithelial loss, and inflammatory infiltration. Since this was a non‐parametric analysis, the data were expressed as medians, and the minimum and maximum limits of the determinations for each experimental group were presented.

For morphometric analysis, villus height (measured from the tip to the villus crypt junction) and crypt depth were quantified in 10 villi and 10 crypts per field across six microscopic fields per group. The villus to crypt ratio was then calculated. The analysis was performed in a blind manner and the results were expressed as mean ± standard error of the mean. The most effective dose of ISO for the management of intestinal mucositis was determined through a comprehensive assessment of histological alterations.

##### 2.2.3.2 Mast Cells and Goblet Cells

For the identification of mast cells and goblet cells, paraffin embedded jejunal samples from the vehicle, ISO‐100 and 5‐FU groups were selected for histological staining. Mast cells were stained with toluidine blue, while goblet cells were visualized using the periodic acid–Schiff (PAS) stain. The determination of mast cells and goblet cells number was performed using an optical microscope equipped with a digital imaging system (LEICA, Wetzlar, Germany). The micrographs were acquired for subsequent analysis and, for each experimental group, these cells were counted in at least 10 fields using Image J software (National Institute of Mental Health, Bethesda, MD, USA). The results were presented as the average number of mast cells or goblet cells/field ± standard error of the mean.

##### 2.2.3.3 Measurement of GSH and MDA Levels

To determine reduced glutathione levels (GSH), 100 µL of the supernatant from a 10% (w/v) homogenate prepared with jejunal segment (tissue/EDTA at 0.02 mol/L) was mixed with 20 µL of 50% (w/w) trichloroacetic acid (TCA) and 80 µL of deionized water. The mixture was vortexed for 3 min and then centrifuged at 3000 rpm for 15 min. After this step, 100 µL of the supernatant was added to another microtube containing 100 µL of TRIS‐HCl buffer (0.4 mol/L, pH 8.9), followed by the addition of 10 µL of 0.01 mol/L DTNB. The reaction medium was kept in a dark place, protected from light, for 10 min. Finally, the absorbance was measured at 412 nm [22].

The levels of MDA, the main reactive substance to thiobarbituric acid were determined by mixing 100 µL of the supernatant from a 10% (w/v) homogenate prepared with jejunal segment (tissue/KCl at 0.15 mol/L) with 750 µL of 1% H_3_PO_4_ and 250 µL of 0.6% thiobarbituric acid (TBA). The mixture was heated at 100°C for 1 h. After this period, the microtubes were cooled in an ice bath, and then 1 mL of n‐butanol was added. The mixtures were vortexed for 1 min and centrifuged at 2000 rpm for 10 min. After this, 100 µL of the supernatant were collected and the absorbance was measured at 535 nm [23].

##### 2.2.3.4 MPO activity

Myeloperoxidase (MPO) activity was determined according to the principles described by Bradley et al. [24], slight modifications. Briefly, jejunal segment (50–100 mg) from the vehicle, ISO (100 mg/kg), and 5‐FU group were homogenized in 1 mL of potassium phosphate buffer containing 0.5% hexadecyltrimethylammonium bromide (HTAB) and then centrifuged at 4000 rpm for 7 min at 4°C to obtain the supernatant for analysis. MPO activity was determined spectrophotometrically by measuring the absorbance at 450 nm after the reaction with o‐dianisidine dihydrochloride and 1% H_2_O_2_. Enzymatic activity was expressed as MPO units per milligram of tissue.

##### 2.2.3.5 Immunohistochemistry for COX‐2

Antigen retrieval was then carried out using citrate buffer (pH 7.0; DAKO, São Paulo, Brazil) for 20 min in a 95°C water bath, using jejunal tissue sections. The slides were rinsed with phosphate‐buffered saline (PBS) (5 min, at 25% ± 2°C) to remove residual reagents. Endogenous peroxidase activity was blocked using 3% H_2_O_2_ solution for 30 min. The sections were subsequently incubated overnight with a goat anti‐COX‐2 primary antibody (Santa Cruz, Dallas, TX, USA) at a 1:100 dilution in antibody diluent. Following PBS washes, a rabbit IgG secondary antibody (GBI Labs, Bothell, WA, USA), diluted 1:400, was applied for 30 min. Visualization of the immunoreaction was achieved by incubating the sections with a streptavidin‐peroxidase complex (ABC system) for 30 min, followed by chromogenic development with 3,3‐diaminobenzidine (DAB; DAKO, São Paulo, Brazil). Counterstaining was performed with hematoxylin (DAKO, São Paulo, Brazil) for 10 min. Negative control slides were processed in parallel under identical conditions, except that the primary antibody was replaced with antibody diluent.

For the analysis of COX‐2 immunostaining, the percentage of positively immunolabeled area was quantified using ImageJ and all images were acquired with an optical microscope equipped with a digital imaging system (LEICA, Wetzlar, HE, Germany), ensuring consistent illumination and magnification across all samples.

##### 2.2.3.6 Statistical Analysis

The normality of the data was analyzed using the Shapiro‐Wilk test. Parametric variables were analyzed using one‐way ANOVA followed by Tukey's post‐hoc test, while nonparametric data, as well as histopathological scores were analyzed using the Kruskal‐Wallis test followed by Dunn's post‐hoc test. A 95% confidence interval was adopted, thus comparisons with *p* < 0.05 were considered significant. Outlier analysis was conducted using the Grubbs' test. Continuous variables were expressed as mean ± standard error of the mean, while histopathological scores were presented as median and range. The statistical tests were performed using GraphPad Prism version 6.0.

## 3 Results and discussion

### 3.1 Histopathological Study

As summarized in Table 2, administration of 5‐FU caused evident mucosal injury, characterized by a reduction in villus height, crypt necrosis, epithelial vacuolization, and pronounced inflammatory infiltration. These alterations resulted in significantly elevated histopathological scores compared with the vehicle group across all intestinal segments (*p* < 0.05). Treatment with ISO at 100 mg/kg markedly reduced the microscopic injury scores relative to the 5‐FU group. In parallel, ISO at doses of 10 or 50 mg/kg did not produce significant histological improvement in any intestinal region (*p* > 0.05).

**TABLE 2:** Histopathological scores in mice treated with ISO with intestinal mucositis induced by 5‐FU.

Intestinal segment	Groups				
	Vehicle	5‐FU	ISO‐10	ISO‐50	ISO‐100
Duodenum	0 (0–1)	3 (2–3)^a^	2 (1–3)	2 (1–2)	1 (1–3)^b^
Jejunum	0 (0–1)	3 (1–3)^a^	2 (1–3)	1.5(1–3)	1 (1–2)^b^
Ileum	0 (0–0)	3 (1–3)^a^	3 (1–3)	2 (1–3)	2 (1–3)

Where

^a^ p < 0.05 vs vehicle group and;

^b^ p < 0.05 vs 5‐FU group using Kruskal–Wallis followed by Dunn's test. 5‐FU: 450 mg/kg, i.p.; ISO‐10: ISO at 10 mg/kg, p.o.; ISO‐50: ISO at 50 mg/kg, p.o.; ISO‐100: ISO at 100 mg/kg, p.o.

**FIGURE 1:**
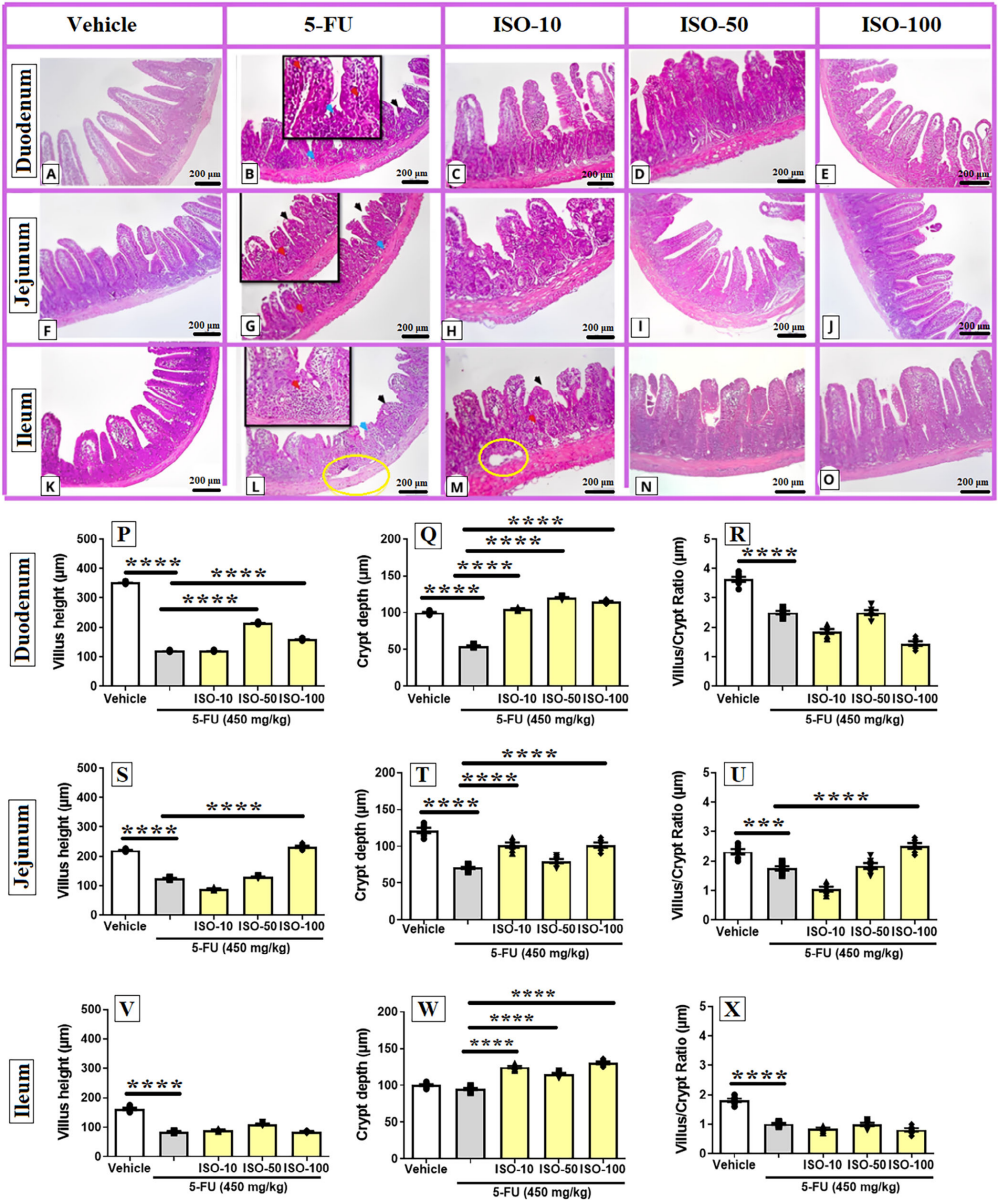
Histopathological evaluation of the protective effect of isoquercitrin (ISO) in intestinal segments (A–O). Black arrows identify villus shortening; red arrows indicate inflammatory cell infiltration; blue arrows highlight disorganization of crypt architecture; and yellow circles denote areas of tissue edema. Morphometric analysis of intestinal segments from mice subjected to 5‐FU–induced intestinal mucositis (P–X). Where, measurements of height of villus in the duodenum, jejunum, and ileum are shown in P, Q and R, respectively. Crypt depth in the duodenal, jejunal, and ileal segments are shown in S, T, and U, respectively. Villus/crypt ratio in the duodenal, jejunal, and ileal segments are shown in V, W and X, respectively. Data are expressed as mean ± SEM. *p < 0.05, **p < 0.005, ***p < 0.0005 and ****p < 0.0001 according to one‐way ANOVA followed by Tukey's test.

Administration of 5‐FU produced a significant elevation in histopathological scores compared with the vehicle group, confirming the severity of the mucosal injury. Treatment with ISO at 100 mg/kg markedly reduced these scores in the duodenal and jejunal segments, showing a statistically significant improvement relative to the 5‐FU group (*p* < 0.05). Figure 1A–O shows the micrographs obtained in the histopathological analysis for H&E staining.

Treatment with 5‐FU caused a marked reduction in villus height across all intestinal segments (duodenum: 120.3 ± 1.2 µm; jejunum: 125.3 ± 1.5 µm and ileum: 85.4 ± 1.5 µm) (Figure 1A–E) when compared with the vehicle group (duodenum: 352.7 ± 1.4 µm; jejunum: 221.0 ± 1.9 µm; and ileum: 162.5 ± 3.8 µm). Administration of ISO at 50 and 100 mg/kg significantly counteracted the villus shortening observed in the duodenum (ISO‐50: 215.0 ± 1.5 µm and ISO‐100: 159.9 ± 1.4 µm) and jejunum (ISO‐50: 130.3 ± 1.4 µm and ISO‐100: 232.5 ± 3.8 µm) (*p* < 0.05), demonstrating partial preservation of mucosal architecture. In the ileum, however, none of the tested doses of ISO (ISO‐10: 90.7 ± 1.7 µm; ISO‐50: 111.2 ± 2.0 µm and ISO‐100: 85.4 ± 1.5 µm) produced a statistically significant recovery in villus height (Figure 1P).

Our findings showed that 5‐FU markedly reduced crypt depth in the duodenal segment (55.8 ± 0.7 µm) compared with the vehicle group (100.0 ± 0.9 µm) (Figure 1F–J). The morphological changes caused by the administration of 5‐FU, such as villus and crypt atrophy, result from increased necrosis of the intestinal mucosa due to its cytotoxic effect, which is already well documented in the literature [2, 4, 7]. These findings support the search for new antineoplastic drugs for chemotherapy, aiming for more effective treatment with fewer side effects [25].

The administration of ISO (ISO‐10: 105.0 ± 0.8 µm; ISO‐50: 120.0 ± 0.9 µm; and ISO‐100: 115.0 ± 0.9 µm) effectively prevented this reduction, maintaining crypt morphology similar to that observed in vehicle group (*p* < 0.05 vs. 5‐FU) (Figure 1Q). In the jejunal tissue, treatment with 5‐FU (70.8 ± 1.6 µm) led to a marked reduction in crypt depth when compared with vehicle group (121.7 ± 3.4 µm). However, the treatment with ISO (ISO‐10: 101.7 ± 3.4 µm; ISO‐50: 80.0 ± 2.7 µm; and ISO‐100: 101.7 ± 3.5 µm) reversed this alteration (Figure 1T). In the ileal segment, the treatment with ISO (ISO‐10: 125.0 ± 1.5 µm; ISO‐50: 115.2 ± 1.5 µm; and ISO‐100: 130.7 ± 1.7 µm) enhanced crypt depth when compared to the data obtained for 5‐FU group (95.0 ± 1.4 µm) (Figure 1W).

The villus/crypt ratio in the duodenum was significantly reduced following 5‐FU administration (2.50 ± 0.05 µm) compared with the vehicle group (3.63 ± 0.08 µm) (Figure 1R). None of the ISO treatment regimens produced a significant increase in this ratio relative to the 5‐FU group (ISO‐10: 1.87 ± 0.08 µm; ISO‐50: 2.50 ± 0.08 µm; and ISO‐100: 1.45 ± 0.08 µm). In the jejunum, 5‐FU (1.75 ± 0.08 µm) also caused a marked decline in the villus/crypt ratio compared with vehicle group (2.32 ± 0.09 µm) (Figure 1U); however, treatment with ISO at 100 mg/kg (2.52 ± 0.09 µm) effectively counteracted this effect. In the ileum, 5‐FU similarly (1.01 ± 0.03 µm) reduced the villus/crypt ratio compared with the vehicle group (1.81 ± 0.05 µm), yet none of the ISO doses tested (ISO‐10: 0.85 ± 0.03 µm; ISO‐50: 1.00 ± 0.05 µm µm and ISO‐100: 0.81 ± 0.06 µm) produced a statistically significant improvement in this parameter (Figure 1X).

The administration of ISO, especially at 100 mg/kg (p.o.), restored villus height and epithelial integrity, while attenuating crypt necrosis and inflammatory cell infiltration in both the duodenum and jejunum. In another study, ISO administration was able to attenuate ischemia/reperfusion injury at the intestinal level by suppressing inflammasome‐mediated inflammation [26], and these events are closely related to tissue necrosis.

The bioactivity of ISO in different injury models of the gastrointestinal tract is associated with their effects on maintaining mucosal integrity, which is a consequence of their cytoprotective effects that are essential for homeostasis in this microenvironment. Furthermore, the modulation of acute inflammation mechanisms has been linked to this effect [27].

The protective effect of ISO against the 5‐FU‐induced intestinal mucositis in mice was highlighted in the jejunum. Therefore, this segment was chosen for further investigation, through the quantification of mediators linked to oxidative stress and inflammation, morphometric analysis and study of the role of COX‐2 expression by immunohistochemistry.

### 3.2 Mast Cells and Goblet Cells

**FIGURE 2:**
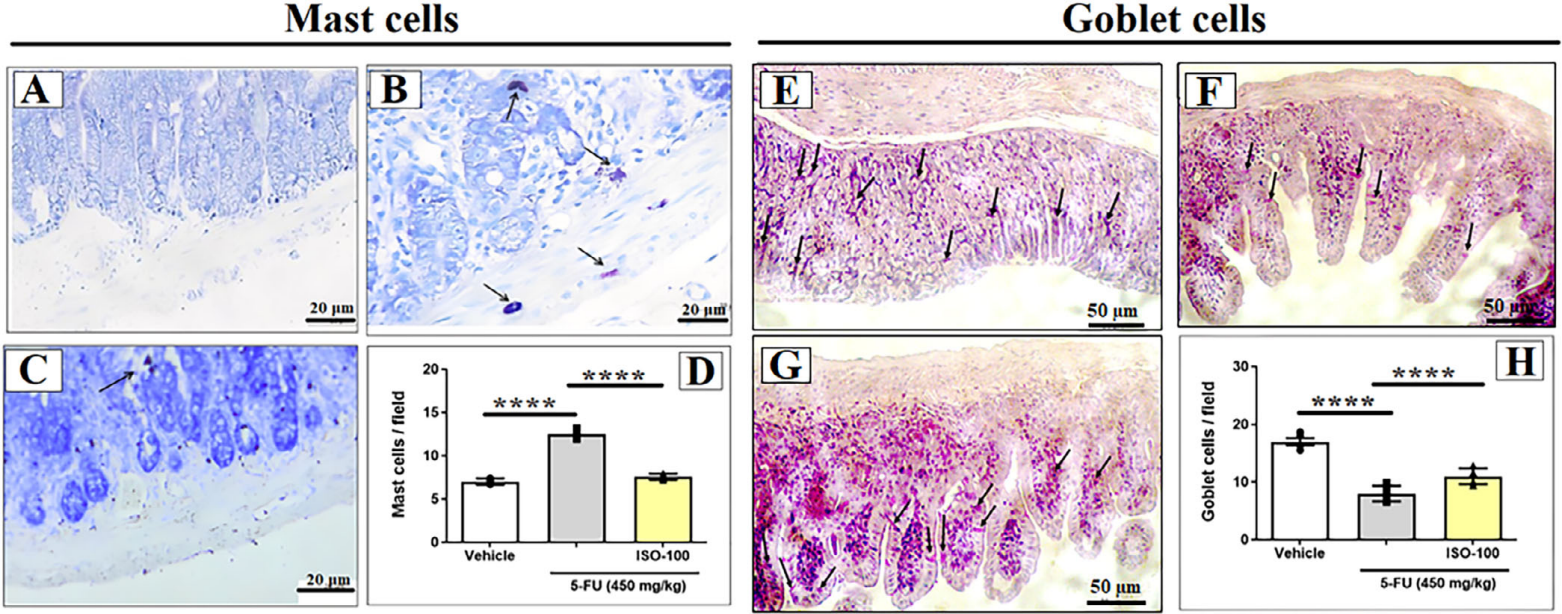
Determination of the number of goblet cells and mast cells in the intestinal mucosa of mice subjected to 5‐FU‐induced intestinal mucositis. Where, (A, E) vehicle group, (B, F) 5‐FU group, (C, G) ISO‐100, (D) statistical analysis of the number of mast cells and (H) statistical analysis of the number of goblet cells. Data are shown as mean ± SEM. *p < 0.05, **p < 0.005, ***p < 0.0005, and ****p < 0.0001 according to one‐way ANOVA followed by Tukey's test.

To determine whether ISO pretreatment modulated the mast cell expansion caused by 5‐FU, mast cells within the intestinal mucosa were quantified, as shown in Figure 2A–C. Treatment with 5‐FU (12.5 ± 0.2 mast cells/field) resulted in a significant increase in mast cell density per microscopic field compared with the vehicle group (7.0 ± 0.1 mast cells/field). The treatment with ISO at 100 mg/kg (7.6 ± 0.1 mast cells/field) markedly reduced the number of mast cells, effectively preventing the mastocytosis and degranulation triggered by 5‐FU in the intestinal tissue of mice (Figure 2D).

Examination of the jejunal segment (Figure 2E–G) revealed that mice receiving 5‐FU (8.0 ± 0.6 cells/field) exhibited a marked decrease in goblet cell numbers within the intestinal mucosa when compared with the vehicle group (17.0 ± 0.5 cells/field). Conversely, pretreatment with ISO at 100 mg/kg (11.0 ± 0.5 cells/field) preserved the number of goblet cells, maintaining values significantly higher than those observed in the 5‐FU group (Figure 2H).

Mast cell degranulation leads to the release of histamine, an important mediator for the vasodilation process and, consequently, increased recruitment of leukocytes to the injured environment [28], while goblet cells produce mucus, which acts as a physical barrier protecting the intestinal epithelium from harmful agents [3, 29]. The reduction in the number of mast cells with ISO administration indicates a reduction in inflammation in the microenvironment, as well as the increase in goblet cells demonstrates the preservation of the intestinal microstructure.

### 3.3 Oxidative Stress and Inflammatory Markers

**FIGURE 3:**
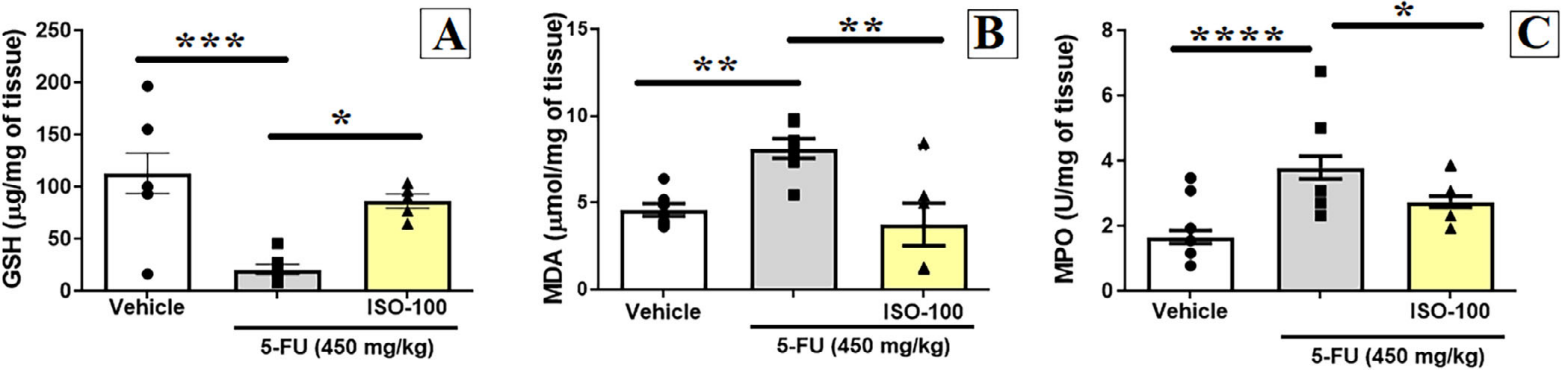
Effect of isoquercitrin (ISO) on oxidative stress and inflammatory parameters in mice with 5‐fluorouracil induced intestinal mucositis: (A) GSH levels, (B) MDA levels, and (C) MPO activity. Data are expressed as mean ± SEM. *p < 0.05, **p < 0.005, ***p < 0.0005 and ****p < 0.0001 according to one‐way ANOVA followed by Tukey's test.

As illustrated in Figure 3A, 5‐FU treatment (20.5 ± 4.9 µg/mg of tissue) caused a significant depletion of GSH content compared with vehicle group (112.8 ± 19.5 µg/mg of tissue). ISO at 100 mg/kg (86.1 ± 6.9 µg/mg of tissue) effectively prevented this reduction, restoring GSH levels close to those compared with the vehicle group, which showed statistical significance (*p* < 0.05). In parallel, 5‐FU administration (8.1 ± 0.6 µmol/mg of tissue) significantly increased MDA concentrations in the jejunal tissue compared with the vehicle group (4.6 ± 0.4 µmol/mg of tissue). However, treatment with ISO at 100 mg/kg (3.7 ± 1.2 µmol/mg of tissue) markedly reduced MDA levels relative to the 5‐FU group (p < 0.05), indicating attenuation of 5‐FU–induced oxidative damage (Figure 3B).

Conversely, as shown in Figure 3C, treatment with 5‐FU (3.8 ± 0.4 U/mg of tissue) produced a significant increase in MPO activity per milligram of tissue compared with the vehicle group (1.6 ± 0.2 U/mg of tissue). The treatment with ISO at 100 mg/kg (2.7 ± 0.2 U/mg of tissue) markedly reduced MPO activity when compared to the 5‐FU group (*p* < 0.05) (Figure 3C).

MDA is the end product of lipid peroxidation, a process in which membrane lipids participate in a chain reaction involving the formation of radical species [30]. On the other hand, GSH is a tripeptide (γ‐glutamyl‐cysteinylglycine) that, due to the presence of a sulfhydryl group (–SH), exhibits a high nucleophilic character, being able to inhibit ROS/RNS. The reduction of MDA levels, combined with the restoration of GSH levels and/or other endogenous antioxidants is an important mechanism associated with the protective effect of natural products against intestinal mucositis, since chemotherapy treatment is responsible for increasing oxidative stress [14].

These data confirms that ISO attenuates intestinal oxidative stress, in line with studies showing restoration of GSH levels and a decrease in MDA levels in preclinical models where inflammation plays a key role [31]. Similarly, in a dextran sulfate sodium‐induced colitis model, ISO administration (1 or 10 mg/kg/day) showed a protective effect by reducing oxidative stress and inflammatory parameters [32]. In addition, the reduction in MPO activity, an enzyme released during neutrophil degranulation, indicates a reduction in leukocyte chemotaxis, which is consistent with the reduction in the number of mast cells, since increased histamine release is an important factor in this process during acute inflammation [33].

### 3.4 Role of COX‐2

Cyclooxygenases are isoenzymes responsible for converting arachidonic acid into prostaglandins (PGs) and thromboxanes (TXs), which are eicosanoids responsible for different functions in the intestinal mucosa, especially their role in regulating inflammatory mechanisms and metabolic adaptations [34, 35]. Patients undergoing cancer treatment present elevated levels of COX and other mediators linked to inflammation and oxidative stress. Interestingly, modulating the levels of these markers is linked to a better prognosis, especially with regard to reducing COX‐2 expression [36].

**FIGURE 4:**
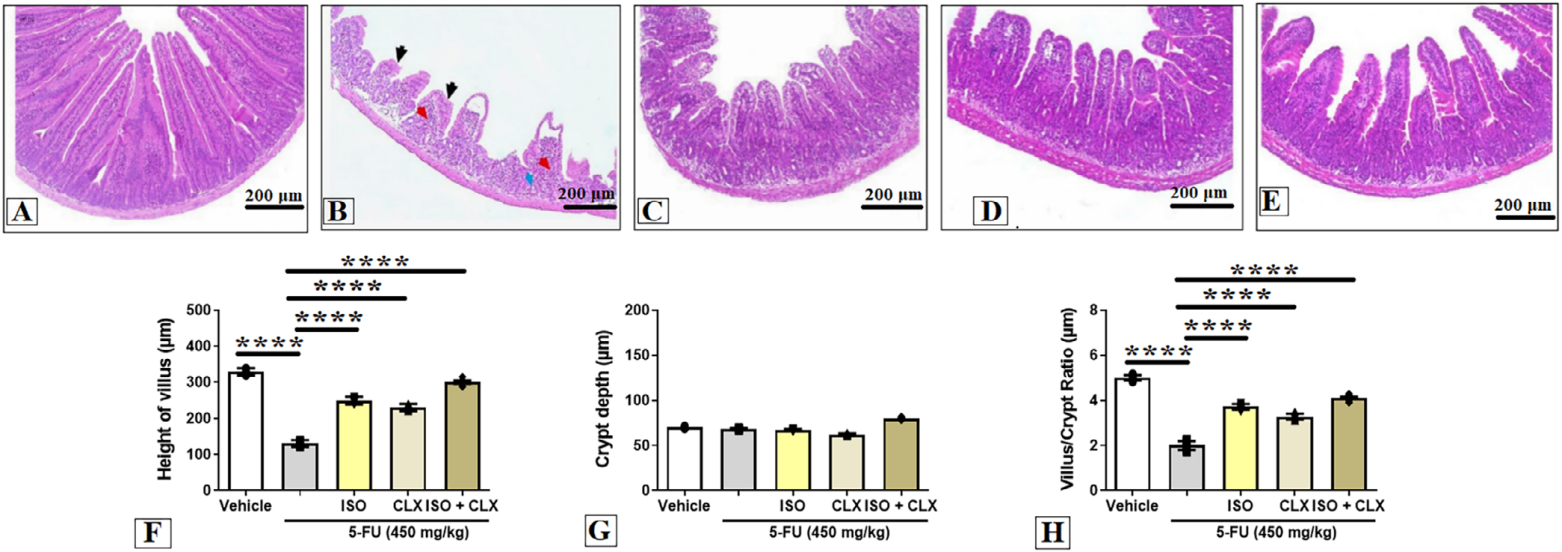
Role of COX‐2 pathway in the effect of ISO against 5‐FU–induced intestinal mucositis. Where, (A) vehicle group, (B) 5‐FU group, (C) ISO‐100, (D) CLX at 7.5 mg/kg, (E) ISO + CLX at 7.5 mg/kg. Statistical analysis for villus height (F), crypt depth (G), and villus/crypt ratio (H). Data are shown as mean ± SEM. *p < 0.05, **p < 0.005, ***p < 0.0005, and ****p < 0.0001 according to one‐way ANOVA followed by Tukey's test.

When analyzing the villus height in the jejunal segment (Figure 4), treatment with 5‐FU (131.0 ± 3.4 µm) resulted in a significant reduction in villus height compared with the vehicle group (329.4 ± 3.6 µm). The treatment with ISO at 100 mg/kg (249.6 ± 3.8 µm) effectively reversed this reduction, restoring villus height to values comparable to the vehicle group.

Treatment with the celecoxib (CLX) (230.4 ± 3.6 µm) significantly prevented the villus shortening induced by 5‐FU. In addition, the group receiving both ISO at 100 mg/kg and CLX (300.6 ± 4.6 µm) showed even greater preservation of the mucosal architecture, with villus height remaining substantially closer to normal compared with the 5‐FU group. This outcome suggests that ISO, when combined with CLX, produces an enhanced protective effect. The group receiving the combined treatment of ISO at 100 mg/kg and CLX exhibited a more pronounced recovery of villus height compared with the groups treated with ISO or CLX alone.

In the analysis of crypt depth in the jejunal segment (Figure 4), no significant difference was observed between the 5‐FU (68.0 ± 0.5 µm) and vehicle (70.0 ± 0.5 µm) groups. Similarly, treatment with ISO at 100 mg/kg (67.0 ± 0.5 µm), CLX (62.0 ± 0.5 µm), and ISO‐100 + CLX (80.0 ± 0.5 µm) did not produce significant changes in crypt depth when compared with the 5‐FU group (*p* > 0.05).

In the evaluation of the villus/crypt ratio in the duodenal segment (Figure 4), treatment with 5‐FU (2.0 ± 0.1 µm) resulted in a significant reduction in this parameter compared with the vehicle group (5.0 ± 0.1 µm). The treatment with ISO (100 mg/kg) (3.7 ± 0.1 µm), CLX (3.2 ± 0.1 µm) or the combination of both agents (4.1 ± 0.1 µm) significantly increased the villus/crypt ratio relative to the 5‐FU group (*p* < 0.05). Moreover, animals receiving the combined treatment of ISO and CLX exhibited a greater restoration of the villus/crypt ratio than those treated with ISO alone (*p* < 0.05), suggesting a potentiated protective interaction between the two treatments.

Histopathological examination of the photomicrographs in Figure 4 (H&E staining) revealed that treatment with 5‐FU caused marked mucosal injury, characterized by villus shortening, disruption of villus‐crypt architecture, presence of edema, and intense inflammatory infiltration when compared with the vehicle group. In contrast, animals pretreated with ISO at 100 mg/kg and/or CLX exhibited attenuation of these histopathological alterations, demonstrating a visible reduction in the tissue damage induced by 5‐FU.

The COX enzyme has two main isoforms, COX‐1 and COX‐2. The COX‐1 is a constitutive isoform in different tissues, such as the stomach, intestines, platelets, kidneys, and liver. This isoform promotes the formation of PGI2, which is responsible for increasing mucus and bicarbonate production, as well as TXA2, which increases platelet aggregation and is involved in the hemostasis process [37].

On the other hand, COX‐2 is an induced isoform, although constitutive in the heart, whose synthesis is increased during inflammation, making it a marker of this process. PGE2 is the main prostaglandin synthesized by COX‐2, and its reduction is linked to protective effects on the gastrointestinal mucosa [38]. In evaluating the protective effect of carvacrol against irinotecan‐induced intestinal mucositis, it was observed that the reduction in COX‐2 levels was associated with a reduction in NF‐κB expression, as well as an increase in the release of anti‐inflammatory mediators, such as the cytokine IL‐10 [12]. Similar effects were observed in the study of the protective potential of nanoencapsulated quercetin against intestinal mucositis induced by 5‐FU, in which, like ISO, this other flavonoid was able to attenuate oxidative stress and improve tissue microarchitecture [39].

**FIGURE 5:**
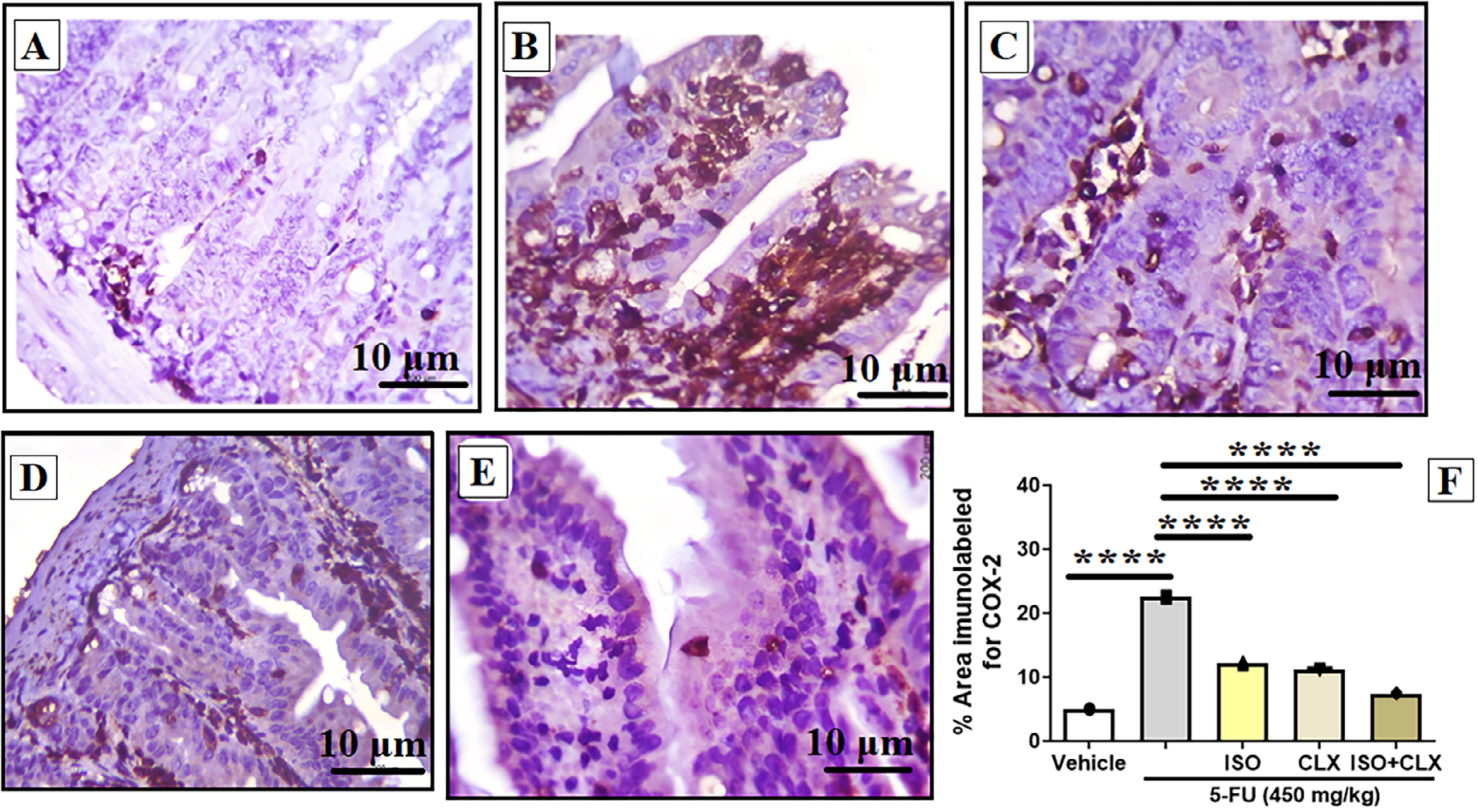
COX‐2 expression in the intestinal mucosa of mice with 5‐fluorouracil–induced intestinal mucositis. Where, (A) vehicle group, (B) 5‐FU, (C) ISO‐100, (D) CLX at 7.5 mg/kg, (E) ISO + CLX at 7.5 mg/kg (F) and quantification of the COX‐2–positive immunolabeled area. Data are presented as mean ± SEM. *p < 0.05, **p < 0.005, ***p < 0.0005, and ****p < 0.0001 according to one‐way ANOVA followed by Tukey's test.

Immunohistochemical evaluation of COX‐2 expression revealed that treatment with 5‐FU induced intense COX‐2 immunostaining (22.5% ± 0.1%) in the intestinal mucosa compared with the vehicle group (5.0% ± 0.1%) (Figure 5). The treatment with ISO at 100 mg/kg markedly reduced COX‐2 immunoreactivity (12.2% ± 0.1%) when compared to the 5‐FU group. Likewise, treatment with CLX alone (11.1% ± 0.1%) or in combination with ISO (7.4% ± 0.1%) further decreased COX‐2 expression compared to the 5‐FU group, demonstrating that ISO modulates inflammatory signaling through COX‐2 inhibition.

The results observed in this study for ISO are supported by evidence that other natural products, including flavonoids, exhibit a protective effect on the intestinal mucosa through the inhibition of COX‐2, and consequently, anti‐inflammatory effects [36, 40]. However, it is estimated that these protective effects are partial COX‐2‐dependent, since other pathways linked to inflammation may be associated with the different pathophysiological events involved in the establishment and progression of chemotherapy‐induced mucositis, such as MAP kinases (MAPK) and iNOS modulation [41, 42].

The MAPK pathway plays a central role in cell proliferation and differentiation processes, as well as in the response to stress and inflammation. Studies indicate that treatment with 5‐FU leads to the activation of p38 MAPK, which is responsible for coordinating the cellular response to stress and inflammation [43]. The p38 MAPK is involved in the regulation of caspase 8, which is responsible for activating the apoptosis process. In the context of mucositis, p38 suppression is responsible for promoting the maintenance of intestinal mucosal integrity due to an anti‐apoptotic effect [41].

iNOS promotes the conversion of arginine to citrulline, which in turn promotes the release of nitric oxide (NO). This mediator plays a dual role during the inflammatory process, being associated with vasodilation, which increases the supply of nutrients and oxygen to the injured microenvironment, but when in excess, it leads to increased nitrosative stress, since its reaction with reactive oxygen species produces peroxynitrite ions [12, 32].

Nonetheless, additional studies are required to elucidate the molecular signaling pathways through which ISO exerts its protective actions particularly those related to pro‐inflammatory cytokine regulation and to further explore other potential mechanisms contributing to its efficacy against chemotherapy‐induced intestinal mucositis.

## 4 Conclusion

Herein, the treatment with ISO substantially alleviated the intestinal injury caused by 5‐FU, leading to notable improvements in tissue architecture, reductions in oxidative imbalance, and diminished recruitment of inflammatory cells. The compound decreased MDA concentrations and MPO activity, enhanced GSH levels, limited mast cell accumulation, and prevented goblet cell loss, thereby preserving villus–crypt morphology and overall mucosal integrity. The present findings indicate that the protective action of ISO involves both direct and indirect modulation of the COX‐2 pathway, as evidenced by the reduction in COX‐2 immunoreactivity. Thus, these results indicate that ISO is a flavonoid capable of attenuating the deleterious effects associated with chemotherapy on the intestinal mucosa, making it a promising natural product for adjuvant therapy of this condition.

## Author Contributions

**Lázaro de Sousa Fideles**: conceptualization, data curation, formal analysis, methodology, writing – original draft, and writing – review and editing. **Matheus da Silva Campelo**: formal analysis, methodology and writing – original draft, writing – review and editing. **João Francisco Câmara Neto**: formal analysis and methodology. **Conceição da Silva Martins**: formal analysis and methodology. **João Erivan Façanha Barreto**: formal analysis and methodology. **ícaro Gusmão Pinto Vieira**: Formal analysis and methodology. **Nágila Maria Pontes Silva Ricardo**: acquisition of funding, resources, writing – review and editing. **Maria Elenir Nobre Pinho Ribeiro**: Conceptualization, acquisition of funding, resources, writing – review and editing.

## Funding

This work was funded in part by the Coordination for the Improvement of Higher Education Personnel—Brazil (CAPES)—N° AUXPE: 1227/2020 and National Council for Scientific and Technological Development—Brazil (CNPq, M.E.N.P.R., research grant N° 307756/2025‐4).

## Ethics Statement

The study was conducted in accordance with the animal research protocol of the Ethics Committee on Animal Use (CEUA) of the Federal University of Ceará (UFC) and was approved under protocol no. 1010230921 on December 7, 2021.

## Conflicts of Interest

The authors declare no conflicts of interest.

## Supporting information




**Supporting File 1**: cbdv71218‐sup‐0001‐SuppMat.docx

## Data Availability

All data are already available in the manuscript.
