# Supplementary material for: Isoquercitrin Alleviates 5‐Fluorouracil‐Induced Intestinal Mucositis in Mice by Modulating Inflammation and Oxidative Stress
Source: Chem Biodivers. 2026 Apr 23;23:e03827. doi: 10.1002/cbdv.202503827 (PMC13104798; doi:10.1002/cbdv.202503827)
Supplement: Supplementary file 1 — Supporting File 1: cbdv71218‐sup‐0001‐SuppMat.docx [file CBDV-23-e03827-s001.docx]

**Supplementary Material**

Lázaro de Sousa Fideles^1^, Matheus da Silva Campelo^2^, João Francisco Câmara Neto^2^, Conceição da Silva Martins^1^, João Erivan Façanha Barreto^1^, Ícaro Gusmão Pinto Vieira^3^, Nágila Maria Pontes Silva Ricardo^2^, Gilberto Santos Cerqueira^1*^, Maria Elenir Nobre Pinho Ribeiro^2*^

^1^ Center for Studies in Microscopy and Image Processing, Faculty of Medicine, Department of Morphology, Federal University of Ceará, Porangabuçu Campus, ZIP Code 60416-030, Fortaleza, Ceará, Brazil

^2^ Laboratory of Polymers and Materials Innovation, Center of Sciences, Department of Organic and Inorganic Chemistry, Federal University of Ceará, Pici Campus, ZIP Code 60440-900, Fortaleza, Ceará, Brazil

^3^ Technological Development Park, 2977 Humberto Monte Avenue, Federal University of Ceará, Pici Campus, ZIP Code 60440-900, Fortaleza, Ceará, Brazil

**ORCID:**

Lázaro de Sousa Fideles: 0000-0001-5303-9142

Matheus da Silva Campelo: 0000-0003-0286-1974

João Francisco Câmara Neto: 0000-0002-2678-8188

Conceição da Silva Martins: 0000-0001-8710-1856

João Erivan Façanha Barreto: 0000-0003-2971-8481

Ícaro Gusmão Pinto Vieira: 0000-0002-0576-3643

Nágila Maria Pontes Silva Ricardo: 0000-0003-1849-5403

Gilberto Santos Cerqueira: 0000-0001-6717-3772

Maria Elenir Nobre Pinho Ribeiro: 0000-0001-6896-7179

**^*^Corresponding authors**

Gilberto Santos Cerqueira

Email address: [cerqueira@ufc.br](mailto:cerqueira@ufc.br)

Maria Elenir Nobre Pinho Ribeiro

Email address: [elenir.ribeiro@ufc.br](mailto:elenir.ribeiro@ufc.br)

**Methods**

**Isolation of isoquercitrin from *Dimorphandra gardneriana* beans**

*Dimorphandra gardneriana* beans was collected in Crato (Ceará, Brazil) and the aerial parts of the plant were used to confirm its authenticity, in which the exsiccata was deposited in the Prisco Bezerra Herbarium of the Federal University of Ceará under protocol number 32339. From 150 g of dried and ground pods, extraction was carried out by sohxlet using water as a solvent, which resulted in obtaining 8.49 g (5.66%) of crude extract. For the purification procedure, this material was dissolved in 200 mL of deionized water, filtered and centrifuged (6000 rpm/10 min) and the supernatant was dried to obtain the purified dry extract.

Isoquercitrin isolation was performed by semi-preparative chromatography using an LC-10AD HPLC (Shimadzu^®^, Kyoto, Japan) coupled to SPD-M10A Photodiode Array detector (Shimadzu^®^, Kyoto, Japan). Sample preparation was performed dissolving 300 mg of the crude extract in 5 mL of methanol, and injecting 1 mL of the resulting solution into the equipment. The analysis was performed at a wavelength of 350 nm, in which the mobile phase (solvent A: aqueous phosphoric acid buffer solution, pH 2.8; solvent B: acetonitrile; 80/20, v/v) was injected at a flow rate of 7 mL/min. After removing the buffer with ion exchange resin, isolated isoquercitrin with a content of 29.49% was obtained.

**Fourier Transform Infrared Spectroscopy (FTIR)**

The infrared spectrum of isoquercitrin was obtained using a Bruker Vertex 70v spectrometer with KBr pellets. The analysis was performed in the range of 4000 - 500 cm^-1^ with a resolution of 2 cm^-1^.

**Results and discussion**

**Structural analysis of isoquercitrin by FTIR**

The molecular structure of isoquercitrin is shown in Fig. 1(a). Fig. 1(b) shows the FTIR spectrum obtained for isoquercitrin isolated from *Dimorphandra gardneriana* beans to confirm its identity. Between 3650 cm^-1^ and 3020 cm^-1^, a broad and intense band with two peaks can be observed, the first at 3485 cm^-1^ and the second at 3280 cm^-1^, which can be attributed to the stretching of the O-H bond present in the alcohol (OH-aliphatic) and phenol (OH-aromatic) functions. The band with a peak at 2940 cm^-1^ refers to the stretching of the C-H bond of methylene groups (CH_2_). The intense band at 1660 cm^-1^ refers to the stretching of the C=O bond of carbonyl groups.

At 1470 cm^-1^, a medium intensity band is observed that can be attributed to the vibrations of the C=C bond of aromatic groups. The bands at 1370 cm^-1^ and 1290 cm^-1^ refer to the vibrations of the C-OH and C-OH bonds of alcoholic and phenolic groups. Meanwhile, the medium intensity band at 1050 cm^-1^ refers to the stretching of the C-O bond of the ether function. The signals between 870 cm^-1^ and 570 cm^-1^ refer to the substitution of the aromatic ring, as well as the fingerprint region of the conjugated carbohydrate to the aglycone. The assignments described above are in agreement with the data reported in the literature for the characterization of isoquercitrin by FTIR [1,2].


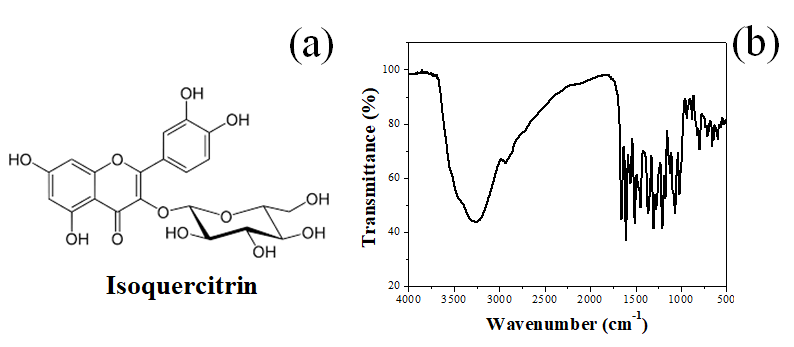


Fig. 1. Molecular structure of isoquercitrin (a). FTIR spectrum obtained for isoquercitrin isolated from *Dimorphandra gardneriana* beans (b).

**References**

[1] Kapoor, M. P.; Moriwaki, M.; Ozeki, M; Timm, D. Structural elucidation of novel isoquercitrin-γ-cyclodextrin (IQC-γCD) molecular inclusion complexes of potential health benefits. Carbohyr. Polym. Tech., 2021, v. 2, p. 100046. http://dx.doi.org/10.1016/j.carpta.2021.100046

[2] Oliveira, R. N.; Mancini, M. C.; Oliveira, F. C. S.; Passos, T. M.; Quilty, B.; Thiré, R. M. S. M.; McGuinness, G. B. FTIR analysis and quantification of phenols and flavonoids of five commercially available plants extracts used in wound healing. Matéria, 2016, v. 21, p. 767-779. http://dx.doi.org/10.1590/s1517-707620160003.0072.
